# Determination of Seroprevalence of Contagious Caprine Pleuropneumonia and Associated Risk Factors in Goats and Sheep Using Classification and Regression Tree

**DOI:** 10.3390/ani11041165

**Published:** 2021-04-19

**Authors:** Abdelfattah Selim, Ameer Megahed, Sahar Kandeel, Abdullah D. Alanazi, Hamdan I. Almohammed

**Affiliations:** 1 Department of Animal Medicine (Infectious Diseases), Faculty of Veterinary Medicine, Benha University, Toukh 13736, Egypt; saharkandeel@fvtm.bu.edu.eg; 2 Department of Animal Medicine (Internal Medicine), Faculty of Veterinary Medicine, Benha University, Moshtohor-Toukh, Kalyobiya 13736, Egypt; ameermegahed@fvtm.bu.edu.eg; 3 Department of Veterinary Clinical Medicine, College of Veterinary Medicine, University of Illinois at Urbana-Champaign, Champaign, IL 61802, USA; 4 Department of Biological Sciences, Faculty of Science and Humanities, Shaqra University, Ad-Dawadimi 11911, Saudi Arabia; 5 Department of Medical Laboratory, Alghad International Colleges for Applied Medical Science, Tabuk 47913, Saudi Arabia; 6 Department of Microbiology and Parasitology, Almaarefa University, Riyadh 11597, Saudi Arabia; hamohammed@mcst.edu.sa

**Keywords:** contagious caprine pleuropneumonia, seroprevalence, decision tree, risk factors, sheep and goats

## Abstract

**Simple Summary:**

The seroprevalence of contagious caprine pleuropneumonia in goats and sheep was determined and the associated risk factors were identified using the Classification and Regression Tree (CART) data mining model. The disease is more prevalent in older animals raised in a flock size ≥200 and that have communal feeding and watering. The CART model showed that the flock size >100 animals is the most important risk factor (importance score = 8.9), followed by age >4 y (5.3) and communal feeding and watering (3.1). The CART model showed more accuracy (area under the curve, AUC = 0.92 than the traditional logistic regression (AUC = 0.89).

**Abstract:**

Classification and Regression Tree (CART) analysis is a potentially powerful tool for identifying risk factors associated with contagious caprine pleuropneumonia (CCPP) and the important interactions between them. Our objective was therefore to determine the seroprevalence and identify the risk factors associated with CCPP using CART data mining modeling in the most densely sheep- and goat-populated governorates. A cross-sectional study was conducted on 620 animals (390 sheep, 230 goats) distributed over four governorates in the Nile Delta of Egypt in 2019. The randomly selected sheep and goats from different geographical study areas were serologically tested for CCPP, and the animals’ information was obtained from flock men and farm owners. Six variables (geographic location, species, flock size, age, gender, and communal feeding and watering) were used for risk analysis. Multiple stepwise logistic regression and CART modeling were used for data analysis. A total of 124 (20%) serum samples were serologically positive for CCPP. The highest prevalence of CCPP was between aged animals (>4 y; 48.7%) raised in a flock size ≥200 (100%) having communal feeding and watering (28.2%). Based on logistic regression modeling (area under the curve, AUC = 0.89; 95% CI 0.86 to 0.91), communal feeding and watering showed the highest prevalence odds ratios (POR) of CCPP (POR = 3.7, 95% CI 1.9 to 7.3), followed by age (POR = 2.1, 95% CI 1.6 to 2.8) and flock size (POR = 1.1, 95% CI 1.0 to 1.2). However, higher-accuracy CART modeling (AUC = 0.92, 95% CI 0.90 to 0.95) showed that a flock size >100 animals is the most important risk factor (importance score = 8.9), followed by age >4 y (5.3) followed by communal feeding and watering (3.1). Our results strongly suggest that the CCPP is most likely to be found in animals raised in a flock size >100 animals and with age >4 y having communal feeding and watering. Additionally, sheep seem to have an important role in the CCPP epidemiology. The CART data mining modeling showed better accuracy than the traditional logistic regression.

## 1. Introduction

Contagious caprine pleuropneumonia (CCPP) is a highly contagious respiratory disease that affects small ruminants, particularly goats, caused by *Mycoplasma capricolum* subsp. *capripneumoniae* (Mccp) [1,2]. The disease is a classical transboundary animal disease, included in the list of notifiable diseases of the World Organization for Animal Health (OIE) [3,4]. Because CCPP is a devastating respiratory infection that causes high morbidity (100%) and mortality (80–100%) as a result of severe fibrinous pleuropneumonia [5], the disease is considered one of the most common respiratory diseases that cause huge economic losses to the goat industry worldwide [6,7]. The disease represents a great threat and mostly infects goats but is also reported as subclinical cases in sheep [8]. The most common clinical signs of CCPP in goats vary between high fever, depression, weakness, loss of appetite, and associated with respiratory manifestations such as cough, dyspnea, and respiratory discharges. Moreover, abortion and high mortality have been reported in some cases [9,10]. According to the OIE, goats with these criteria are defined as CCPP positive if, (1) Mccp is isolated or there is strong serological evidence of Mccp, (2) only lung and pleura are affected (pleuropneumonia), (3) there is an absence of enlargement of the interlobular septa of the lung (OIE, 2008).

According to the information reported by OIE, CCPP has been reported in nearly 40 countries, mostly in Africa and the Middle East where most of the worldwide goat population exists [11,12]. The disease was first identified in Algeria in 1873 [13]. Since then, it has been reported in Turkey in 2003 [14], Iran during 2006–2007, Oman during 2008–2009 [8], and Yemen in 2009 [2,15]. In Egypt, the goat population gets more attention and the Mccp was recently isolated and detected among sheep and goats in Giza Governorate in 2015 [16], and in Matrouh governorate during 2017–2018 [17].

In fact, only 20 countries isolated Mccp because of the fastidious nature of the organism and the massive application of antibiotic treatment in suspected cases [18]. Therefore, serological tests were widely used to detect antibodies against Mccp such as complement fixation test, indirect hemagglutination, and latex agglutination [3,19,20]. Recently, a highly specific cELISA for CCPP has been developed and used on a large scale [21,22]. In Egypt, the sheep and goat population is significantly large and is considered an important source of milk and meat [23]. However, few small-scale studies are conducted to track the seroprevalence of CCPP among small ruminants in Egypt.

Various risk factors, including animal- and environment-related factors, play an important role in the epidemiology of CCPP [24]. The complexities of risk factor interrelationships in a model that predicts CCPP risk are far from clear. Therefore, examining and understanding the risk factors and their interactions may be central to understanding the epidemiology of CCPP and helping in the design and analysis of other related CCPP epidemiological studies. The Classification and Regression Tree (CART) is a machine-learning algorithm that has been utilized in clinical settings as an ideal tool for clinical decision-making and to assess risk factors [25,26]. Despite its potential, the CART has been seldom used in population-based epidemiology and genetic epidemiology studies. The second objective of this study was therefore to use CART modeling to identify risk factors associated with CCPP in Egyptian sheep and goats in a large study population. We believe that the results of this study provide contemporary information about CCPP seroprevalence and associated risk factors that can be used as a guide for future CCPP epidemiological studies.

## 2. Materials and Methods

### 2.1. Description of Study Area

The study area of the serosurvey comprised Kafr ElSheikh, Alexandria, Menofia, and Gharbia Governorates. Geographically, these areas are located between 30.12861 to 31.2057531° N latitude and 29.924526 to 31.242222° E longitude. Additionally, these Governorates have a Mediterranean climate: cool, humid, and with rainfall during winter and warm and dry during summer. These areas are characterized by various agro-climates, which are suitable for different kinds of crop production and the raising of sheep and goats.

### 2.2. Study Design and Sampling

A cross-sectional study was performed during 2019 to determine the seroprevalence and to assess the associated risk factors for CCPP in sheep and goats in four Governorates at the Nile Delta of Egypt. The minimum sample size by Governorate was determined for CCPP seroprevalence of 5% and 32% in sheep and goats during the study period, respectively [16]. The effective sample size by Governorate was approximately 62 sheep and 36 goats to estimate the seroprevalence of 5% and 32% with a precision of ±2.5% (50% relative error) at the 95% confidence level. Within Governorates, the animals were randomly selected from different geographic locations. For each flock, between 1 and 3 animals were randomly selected depending on the size of the flock.

A total of 620 animals (390 sheep and 230 goats) were screened for CCPP. Blood samples were collected from the jugular vein using 20 G needles and 10 mL blood collecting tubes with EDTA. The serum was separated by centrifugation of the blood sample at 3000 rpm/min for 10 min. The serum samples were stored at −20 °C for serological testing. The following information was obtained before blood collection: age, gender, flock size, and communal feeding and watering.

### 2.3. Serological Examination

The antibodies against Mccp were serologically detected using a commercial cELISA kit (IDEXX, Montpellier, France) according to the manufacturer’s instructions. The optical density (OD) was measured using an ELISA microplate reader at 450 nm. The results were interpreted according to the following formula: percent of inhibition (PI) = (OD monoclonal antibodies, Mab − test serum)/(OD Mab − OD conjugate) × 100. The results are considered positive when PI% is greater than or equal to 55%

### 2.4. Statistical Analysis

All statistical analyses were performed with a statistical software program (SAS 9.4, SAS Inst.Inc., Cary, NC, USA). The variables were categorized as follows: dependent variable CCPP (present: 1, absent: 0) and independent variables species (2 levels: sheep and goats), gender (2 levels: male and female), age (5 levels: <1.0, 1.0–<2.0, 2.0–<3.0, 3.0–<4.0, ≥4.0 y), flock size (4 levels: ≤50, 51–100, 101–200, 201–300 animal), and communal feeding and watering (2 levels: yes and no), and geographic location (4 levels: Alex, KF, MF, and Qal). The seroprevalence of CCPP at different levels of proposed risk factors was calculated using PROC FREQ of SAS. The associations between CCPP seroprevalence and proposed risk factors were evaluated using the Cochran–Armitage trend test, and the strength of associations was evaluated using Phi and Cramer’s V value. Univariable and stepwise forward multivariable random effects logistic regression were used to identify the significant risk factor(s) associated with CCPP. A *p*-value of 0.05 was used for entry and exit of the model. In the multivariable logistic regression, we treated the flock size and geographic locations as random effects to account for the potential clustering of animals within flocks and the clustering of flocks within geographic locations, respectively. The final model fit was assessed using the Hosmer–Lemeshow goodness-of-fit test. The strength of the association of independent variables with the outcome variable (CCPP) was expressed as a prevalence odds ratio (POR) with 95% confidence intervals [27].

A data mining technique, CART, was used to show the relationship between important risk factors and their hierarchical classification in the tree diagram visualization. The advantage of the CART model is that it is nonparametric and is suitable for nonlinear structure, making it appropriate for solving complex dynamic clinical problems from a small dataset [28]. Classification trees were used to analyze categorical outcomes. Two-step algorithms were used to build the CART: splitting and pruning. The splitting step is to determine the best variable for splitting using the Gini index by the following equation:

$$I(A)=1-\sum_{k=1\;P_{k}^{2}}^{m}\;$$

where *p_k_* is the proportion of the sample entering the *k* class. A large classification tree was created and to develop a tree with the best size and lowest misclassification rate, a pruning step was used by cross-validation [26]. Receiver Operating Characteristic (ROC) curves and the area under the curve (AUC) were used to assess the classification accuracy. An AUC value of 0.5 indicates that the classification model cannot distinguish between healthy and CCPP animals. AUC values of 0.7 to 0.8 indicate a good classification model, AUC values of 0.8 to 0.9 indicate an excellent classification model, and AUC > 0.9 indicates the perfect classification model [29].

## 3. Results

The seroprevalence of CCPP was determined in 620 serum samples obtained from 390 sheep and 230 goats with an age range from 10 months to 5 years and raised in flock sizes ranging from 15 to 220 animals located in four governorates in the Nile Delta of Egypt (Alex, KF, MF, and Qal). The distribution of animals based on the risk factors is described in Table 1.

**Figure 1:**
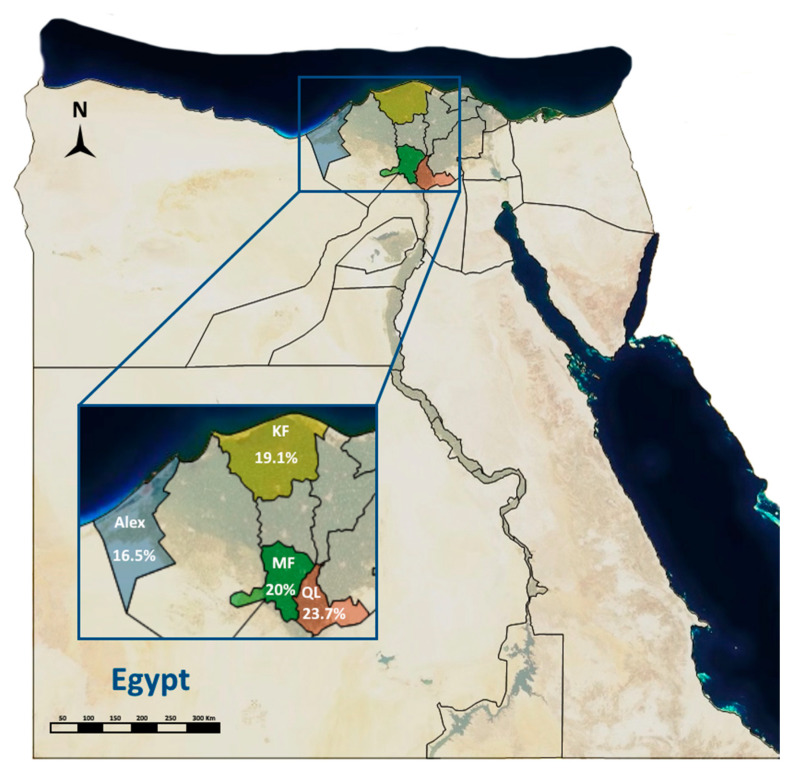
Geographic distribution of contagious caprine pleuropneumonia in sheep and goats of the Nile Delta of Egypt.

The total seroprevalence of CCPP in this study is 25.6% in sheep and 10.4% in goats (total, 20%). The seroprevalence of CCPP differed non-significantly between the localities under study. Qalyoubia Governorate showed the highest seroprevalence of CCPP (23.7%) as shown in Table 1 and Figure 1.

The distribution of CCPP-positive animals differed between species (*p* = 0.004), ages (*p* < 0.001), flock sizes (*p* < 0.001), and for the use of communal feeding and watering (*p* < 0.001; Table 1). The highest seroprevalence of CCPP was present among animals of age >4 y (48.7%) raised in a flock size >200 animals (100.0%) with communal feeding and watering (28.2%).

The results of this study showed strong associations between the seroprevalence of CCPP infection and flock size (Phi Coefficient and Cramer’s V = 0.71), and weak association with species (0.22), age (0.30), and communal feeding and watering (0.30). However, the Phi Coefficient and Cramer’s V of 0.10 and 0.01 indicated no association between CCPP seroprevalence and geographic location and gender, respectively.

**Figure 2:**
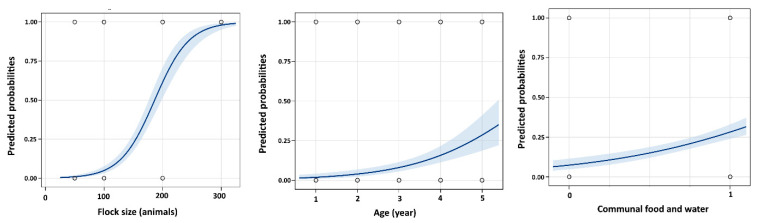
Probability plot for the ability of flock size, age, and communal feeding and watering categories to predict the prevalence of contagious caprine pleuropneumonia (CCPP) in the Nile Delta sheep and goats in Egypt. The curve shows the likely probability of positive CCPP associated with each large category of flock size, age, and communal feeding and watering with the 95% confidence interval shaded blue.

The stepwise logistic regression model indicated that communal feeding and watering, age, and flock size were significant risk factors for CCPP-infected animals (Table 2). The animals raised on communal feeding and watering and older animals showed significantly higher POR of CCPP (POR = 3.7, 95% CI 1.9 to 7.3; POR = 2.1, 95% CI 1.6 to 2.8; respectively; Table 2). Additionally, the animals raised in a flock size ≥300 animals increased the risk (99%) of being seropositive for CCPP (Figure 2).

**Figure 3:**
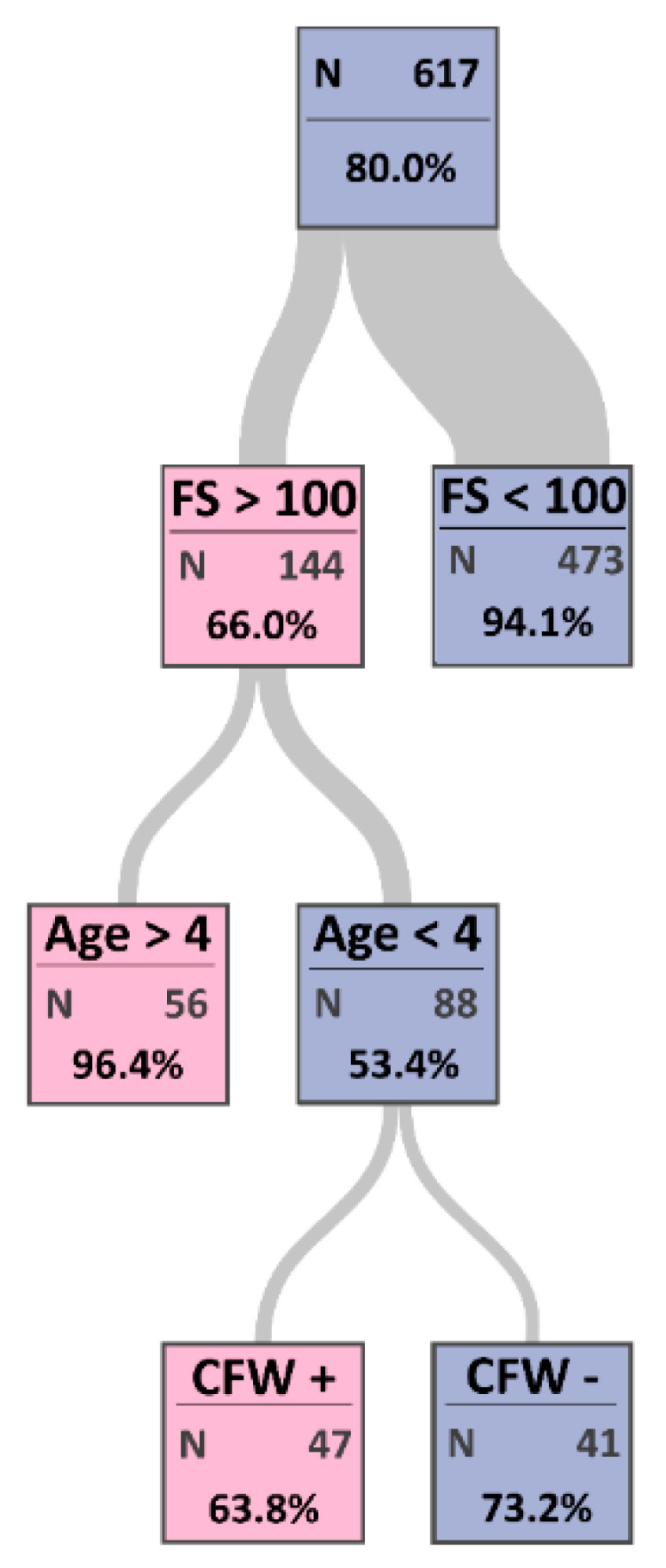
Estimated classification tree determining the most important risk factors of contagious caprine pleuropneumonia (CCPP) in sheep and goats. This tree started with node 0 representing all observations. The color of the square in each leaf node indicates the most frequent level of CCPP among the observations in that node, and this is also the classification level assigned to all observations in that node. The color of the node indicates seronegativity (purple) and seropositivity (pink) for CCPP. Each node has a proportion of observations that have the most frequent level in that node. The width of the link between parent and child nodes is proportional to the number of observations in the child node. The classification tree analysis indicated that flock size (FS), age, and communal food and water (CFW) are the most important risk factors of CCPP.

The CART model developed a decision tree for the most important risk factors of CCPP with maximum certainty (Figure 3) and the misclassification rate was 7.1%. The sensitivity and specificity of the CART model were 97.8% and 73.0%, respectively. The first node in the tree diagram indicates the most important risk factor.

The first node in the tree diagram was the flock size with a cutoff value of 100 animals, where a flock size >100 is the most important risk factor (importance = 8.9; Table 3). The second important risk factor was age with a cutoff value of four years (importance = 5.3; Table 3), and the third node was communal feeding and watering (importance = 3.1; Table 3). CCPP was present for 63.8% of 47 animals that were sharing food and water sources, aged more than four years, and raised in a flock size >100 animals.

In the comparison of the CART with traditional multivariable logistic regression analyses, both approaches had the same results. However, the CART model had better accuracy than the logistic based on the AUC values from both models, where the AUC value of logistic regression was 0.89 (95% CI 0.86 to 0.91), and for the CART was 0.92 (95% CI 0.90 to 0.95). Therefore, based on the CART, CCPP is most likely to be found in animals raised in a flock size >100 animals with age > four years having communal feeding and watering.

## 4. Discussion

It is already well established that CCPP is a highly contagious devastating respiratory disease in sheep and goats, affecting more than 40 countries worldwide, particularly in Africa, Asia, and Middle East [30]. To the best of our knowledge, this is the first large-scale study investigating the CCPP seroprevalence among sheep and goats and the associated risk factors in Egypt. Therefore, the results of this study add substantially to our understanding of CCPP dynamics in the Nile Delta of Egypt and the role of sheep in its epidemiology and are helpful in choosing and creating effective prevention programs. The major strengths of the current study are: (1) a broad context including many geographic locations, placing it amongst the few studies that have examined the seroprevalence of CCPP across the most densely sheep- and goat-populated areas in Egypt; and (2) we used an accurate data-mining technique in an attempt to identify the risk factors and their interactions associated with CCPP in sheep and goats. The main finding of this study based on CART modeling is that the animals raised in a flock size >100 animals with age > four years having communal feeding and watering are at high risk of CCPP. Additionally, sheep seem to have an important role in the population-level dynamics of CCPP. Future research should therefore concentrate on the role of sheep in CCPP epidemiology.

Contagious caprine pleuropneumonia was recently identified in Egypt in 2015 [31]. Here, the antibodies against CCPP were detected in 124 out of 620 (20.0%) examined animals. The reported seroprevalence rate was higher than that reported in Refs. [16,17], where they reported prevalences of 7.2 and 14.2, respectively. However, it concurs with other rates reported in different countries, the seroprevalence of CCPP in Ethiopia was 26% [8], 20–52% in goats and 23–36% in sheep in southern Tanzania [32]. In India, the seroprevalence of CCPP varied widely from 5% [33] to 64% [34]. In eastern Turkey, CCPP seroprevalence was 38% in goats [35]. This marked variability in the CCPP prevalence could arise from differences in species, sample size, sample techniques, and the test used to identify CCPP [9].

In this study, we reported a marked difference in the seroprevalence of CCPP between sheep and goats in agreement with earlier studies [17,32,36,37,38]. However, all these studies reported higher seroprevalence of CCPP in goats than sheep, contrary to the results of this study, where we reported a seroprevalence of 25.6% in sheep and 10.4% in goats, which is considered a new finding. This might be due to sheep being the predominant small ruminant in the Nile Delta of Egypt [39]. The Nile Delta of Egypt is an agricultural area that is more suitable for the grazing and intensive breeding of sheep than goats [40]. Additionally, pastoralism plays an important role in the introduction of the disease to disease-free flocks [41]. In Tanzania, they reported close seroprevalence of CCPP (27.0%) in sheep [2]. However, the pooled CCPP prevalence ranged from 15.74% to 50.0% in sheep and 3.70% to 51.65% in goats depending on the country [2]. Interestingly, the pooled prevalence of CCPP in Africa, Asia, and Southeast Europe was 23.2% in sheep, which is consistent with our findings [2]. In most of the studied areas, sheep kept with goats showed CCPP seropositivity [12]. Therefore, this study supports that sheep have an important role in the CCPP epidemiology.

To the best of our knowledge, this is the first study that determined the risk factors for CCPP in sheep and goats using the CART approach. In this study, the CART and logistic regression identify the same risk factors, however, the CART showed a higher AUC. Classification tree and logistic regression analyses are complementary approaches, but regression analysis relatively focuses on the occurrence of outcomes, while the CART relatively focuses on specificity, where it detects the variables that powerfully predict outcomes for just a subgroup of animals [42]. Classification tree analysis is important for displaying multilevel interactions between variables. Additionally, the CART provides optimal sequential decision rules that are theoretically significant and help in clinical decision-making [42]. In this study, the CART identified the risk factors and correctly classified approximately 99.5% (617/620) of all animals in the dataset. The classification tree supports previous studies using multivariable logistic regression to identify risk factors [2,30,41].

Results of the CART revealed that flock size, age, and communal feeding and watering are the main risk factors of CCPP. Sheep and goats that live in big flocks are at high risk of CCPP. These results are supported by several earlier studies [2,7,30]. The classification tree identified a cut-point of flock size >100 animals as a risk factor for CCPP, which is consistent with an earlier study that reported a flock size of >100 animals to be at high risk of CCPP (OR 2.5; 95% 1.5–3.9). However, Parray and their colleagues in 2019 reported that a flock size of >300 animals is more predisposed to CCPP. On the contrary, various studies reported an insignificant impact of flock size on the CCPP prevalence [12,41]. The reasons behind the higher prevalence of CCPP in large flock sizes are the higher persistence of Mccp in various animals in larger flocks than in smaller flocks and the mixing of infected and uninfected animals [41]. Direct contact is the main way of transmission of CCPP through aerosols, droplets, or nasal discharge [9]. Additionally, pastoralism of large flocks the allows mixing of animals from various flocks, spreading the infection, and this is because intensive management in large flocks is difficult compared to small flocks.

Among the risk factors identified by the CART is age, which is consistent with earlier studies that reported a higher prevalence of CCPP among older animals [12,43,44]. However, this finding also contradicts some earlier studies [10,45,46]. The higher prevalence of CCPP in older animals may be attributed to greater exposure to Mccp over a longer period of time and is not necessarily the result of a new infection [2]. Additionally, reduced physiological status and poor host protection mechanisms in older animals might play an important role in the higher CCPP prevalence compared to younger animals [47]. The results of this study showed that communal feeding and watering is an important risk factor of CCPP. This might be due to the close contact between diseased and healthy animals and, consequently, the circulation of the disease in the population [47]. The main limitation of the CART model was that the clustering of animals within flocks was not considered.

## 5. Conclusions

The results of the present study confirm the circulation of CCPP among sheep and goats in Egypt and provide updated information concerning the geographic area affected by Mccp. Further research should be conducted to assess the study’s outcomes in a longer-term study. Based on accurate CART data mining modeling, older sheep and goats (>4 y) raised in a flock size >100 animals and having communal feeding and watering are at high risk of CCPP.

## Figures and Tables

**Table 1:** Univariable logistic regression analysis of the association of contagious caprine pleuropneumonia in sheep and goats with different risk factors in the Nile Delta of Egypt.

Risk Factors	Category	N	No. Positive	Prevalence (%)	*p*-Value
Geographic location	Alexandria	121	20	16.5	0.138
	Kafr-El-Sheikh	178	34	19.1	
	Menofia	165	33	20.0	
	Qalyoubia	156	37	23.7	
Species	Sheep	390	100	25.6	0.004
	Goat	230	24	10.4	
Flock size	≤50	179	2	1.1	0.001
	51–100	296	26	8.7	
	101–200	123	74	60.2	
	201–300	22	22	100.0	
Communal feeding and watering	Yes	376	106	28.2	0.001
	No	244	18	7.3	
Gender	Male	65	14	21.5	0.743
	Female	555	110	19.8	
Age (years)	<1	110	8	7.2	0.001
	1–<2	155	16	10.3	
	2–<3	132	27	20.5	
	3–<4	142	34	23.9	
	4–5	78	38	48.7	

**Table 2:** Multiple stepwise logistic regression analysis of variables associated with sheep and goats that are positive with contagious caprine pleuropneumonia in the Nile Delta of Egypt.

Variable	Categories	*β*	SE	*p*-Value	POR	95% CI_POR_
Intercept		−9.4	0.8	<0.001	-	-
Flock size	≤50	Reference				
	51–100	0.00	0.00	0.380	0.78	0.38–2.21
	101–200	0.02	0.00	<0.001	1.08	1.02–1.19
	201–300	0.03	0.01	<0.001	1.14	1.03–1.22
Age (years)	<1	Reference				
	1–<2	0.09	0.00	0.987	0.54	0.11–2.05
	2–<3	0.54	0.14	0.083	1.07	0.82–2.32
	3–<4	0.71	0.21	0.004	1.51	1.32–2.24
	4–5	0.76	0.15	<0.001	2.14	1.60–2.84
Communal feeding and watering	No	Reference				
	Yes	1.32	0.34	<0.001	3.68	1.86–7.30

**Table 3:** Important scores of the Classification and Regression Tree for selecting the risk factors of contagious caprine pleuropneumonia.

Variable	Training	
	Relative	Importance
Flock size	1.0000	8.9231
Age	0.5885	5.2947
Communal feeding and watering	0.2744	3.1298

## Data Availability

The datasets used and analyzed during the current study are available from the corresponding author on reasonable request.
